# *CCL3L1-CCR5* Genotype Improves the Assessment of AIDS Risk in HIV-1-Infected Individuals

**DOI:** 10.1371/journal.pone.0003165

**Published:** 2008-09-08

**Authors:** Hemant Kulkarni, Brian K. Agan, Vincent C. Marconi, Robert J. O'Connell, Jose F. Camargo, Weijing He, Judith Delmar, Kenneth R. Phelps, George Crawford, Robert A. Clark, Matthew J. Dolan, Sunil K. Ahuja

**Affiliations:** 1 Veterans Administration Research Center for AIDS and HIV-1 Infection, South Texas Veterans Health Care System, San Antonio, Texas, United States of America; 2 Department of Medicine, University of Texas Health Science Center, San Antonio, Texas, United States of America; 3 Infectious Disease Clinical Research Program, Uniformed Services University, Bethesda, Maryland, United States of America; 4 Infectious Disease Service, Wilford Hall United States Air Force Medical Center, Lackland Air Force Base, Texas, United States of America; 5 Henry M. Jackson Foundation, Wilford Hall United States Air Force Medical Center, Lackland Air Force Base, Texas, United States of America; 6 San Antonio Military Medical Center, Fort Sam Houston, Texas, United States of America; 7 Stratton Veterans Affairs Medical Center, Albany, New York, United States of America; 8 Albany Medical College, Albany, New York, United States of America; 9 Department of Microbiology and Immunology and Biochemistry, University of Texas Health Science Center, San Antonio, Texas, United States of America; University of California San Francisco, United States of America

## Abstract

**Background:**

Whether vexing clinical decision-making dilemmas can be partly addressed by recent advances in genomics is unclear. For example, when to initiate highly active antiretroviral therapy (HAART) during HIV-1 infection remains a clinical dilemma. This decision relies heavily on assessing AIDS risk based on the CD4^+^ T cell count and plasma viral load. However, the trajectories of these two laboratory markers are influenced, in part, by polymorphisms in *CCR5*, the major HIV coreceptor, and the gene copy number of *CCL3L1*, a potent CCR5 ligand and HIV-suppressive chemokine. Therefore, we determined whether accounting for both genetic and laboratory markers provided an improved means of assessing AIDS risk.

**Methods and Findings:**

In a prospective, single-site, ethnically-mixed cohort of 1,132 HIV-positive subjects, we determined the AIDS risk conveyed by the laboratory and genetic markers separately and in combination. Subjects were assigned to a low, moderate or high genetic risk group (GRG) based on variations in *CCL3L1* and *CCR5*. The predictive value of the *CCL3L1-CCR5* GRGs, as estimated by likelihood ratios, was equivalent to that of the laboratory markers. GRG status also predicted AIDS development when the laboratory markers conveyed a contrary risk. Additionally, in two separate and large groups of HIV^+^ subjects from a natural history cohort, the results from additive risk-scoring systems and classification and regression tree (CART) analysis revealed that the laboratory and *CCL3L1-CCR5* genetic markers together provided more prognostic information than either marker alone. Furthermore, GRGs independently predicted the time interval from seroconversion to CD4^+^ cell count thresholds used to guide HAART initiation.

**Conclusions:**

The combination of the laboratory and genetic markers captures a broader spectrum of AIDS risk than either marker alone. By tracking a unique aspect of AIDS risk distinct from that captured by the laboratory parameters, *CCL3L1-CCR5* genotypes may have utility in HIV clinical management. These findings illustrate how genomic information might be applied to achieve practical benefits of personalized medicine.

## Introduction

The last few years have witnessed an unprecedented interest and effort in identifying the genetic determinants that underlie susceptibility to human diseases. Concurrently there is strong interest in developing ways to use this genetic information to provide “individualized medicine”, i.e., tailor the clinical care of patients according to specific elements of their genetic constitution that can convey independent predictive capacity with respect to disease prognostication. However, a framework of how to assess fully whether such genetic information might help improve the clinical management of patients remains unclear, especially when compared to laboratory markers that are considered the gold standard in evaluating disease prognosis. To address this gap in knowledge we used (i) HIV infection, (ii) variations in the genes encoding CC chemokine receptor 5 (CCR5), the major coreceptor for HIV-1 [1], and CC chemokine ligand 3-like 1 (CCL3L1), the most potent CCR5 agonist and HIV-suppressive chemokine [2], [3], [4], [5], and (iii) the laboratory markers (CD4^+^ T cell count and viral load) currently used to evaluate HIV-infected patients, as a model system.

HIV-1-infected subjects are typically started on highly active antiretroviral therapy (HAART) when their CD4^+^ T cell count reaches a threshold below which their risk for developing AIDS increases significantly [6], [7], [8], [9]. These HAART-initiating CD4^+^ T cell count thresholds vary depending on the clinical and economic settings, but are typically between 200 and 350 CD4^+^ T cells/mm^3^ [6], [7], [8], [9]. Nevertheless, when to initiate HAART in HIV-infected subjects remains a clinical dilemma [10], [11], especially when individuals present for clinical care with CD4^+^ T cell counts above 350. Those favoring early initiation cite, among other reasons, the risk that progressive immunologic damage will be incompletely reversible after initiation of HAART [10]. However, there are significant inter-subject differences in the rate at which individuals lose and gain CD4^+^ T cells before and after receipt of HAART, respectively. Consequently, identifying subjects who, despite HAART, are at greater risk of persistent immunologic damage, or predicting how soon an HIV-infected individual might arrive at a predetermined HAART–initiating CD4^+^ T cell count poses a diagnostic challenge. Furthermore, although a CD4^+^ T cell count and plasma viral load provides an excellent snapshot of the immunological and virological status of the infected host at the time of their clinical assessment, they are imperfect surrogates of AIDS risk. This is because both reside downstream of the causal pathways that mediate the extent of CD4^+^ cell loss and viral replication [12], [13], and hence, they do not have any inherent capacity to predict their future trajectory. This necessitates serial determinations of these biomarkers to assess AIDS prognosis.

The trajectories of CD4^+^ T cell counts before and during HAART are likely to be dependent on host-viral interactions [14], [15], [16], [17], [18], [19], [20], [21], [22], [23], [24], [25], [26]. Additionally, during HAART these trajectories are also likely to depend in part on the regenerative capacity of the host as up to 30% of HIV-infected subjects have impaired recovery of CD4^+^ T cell counts, despite suppression of viral replication [27], [28], [29]. Additionally, a large proportion of patients who initiate therapy with a low CD4^+^ nadir (<200 cells/mm^3^) fail to normalize CD4^+^ counts, despite HIV-suppressive HAART [27], [29], [30], [31], [32], [33], [34], [35]. Thus, accounting for polymorphisms in host genes that participate in causal host-viral interactions that affect CD4^+^ T cell depletion before HAART and the immune reconstitution (i.e., recovery of CD4^+^ T cell numbers and function) during HAART might provide a measure of genetic risk that could aid in the clinical assessment and management of HIV-infected subjects. Conceivably, those subjects whose genetic constitution confers a greater risk of progressing rapidly to AIDS as well as impaired recovery during HAART might benefit from earlier initiation of therapy and possibly also from adjuvant therapies that promote immunological recovery (e.g. recombinant IL-7 [36]).

In this study, we focused on evaluating the prognostication capacity of genetic variations in *CCR5* and *CCL3L1* vis-à-vis the laboratory markers for the following reasons. There is substantial data demonstrating that polymorphisms in *CCR5* are associated with variable susceptibility to HIV, AIDS progression rates, and CD4^+^ T cell count recovery during HAART [14], [15], [16], [17], [18], [19], [20], [21], [22], [23], [37], [38], [39], [40], [41]. The copy number of *CCL3L1* influences mRNA/protein expression levels of this potent HIV suppressive chemokine [2], [5] as well as proportion of CD4^+^ T cells expressing CCR5 [5], [42], and *CCL3L1* gene dose has been associated with intersubject differences in (i) susceptibility to HIV acquisition in European-, African-, Hispanic-American adults, intravenous drug users from Estonia and hemophiliacs from Japan [5], [43], [44], as well as children exposed perinatally to HIV-1 from Argentina and South Africa [5], [45], [46]; (ii) viral load in different cohorts, including subjects who were recruited during acute/early infection as well as HIV^+^ women from South Africa [5], [20], [45], [47]; (iii) rates of progression to AIDS in European and African Americans [5]; (iv) HIV-specific CD4^+^ and CD8^+^ T cell responses [47]; and (v) recovery of CD4^+^ T cell counts in subjects who received HAART during chronic or acute/early HIV infection [23]. Additionally, we found that *CCL3L1* copy number and *CCR5* genotypes that were associated with reduced cell mediated immune responses in HIV-negative individuals of European descent were similar to those that were associated with a rapid rate of disease progression in HIV^+^ European Americans [20], suggesting that the CCL3L1-CCR5 axis might play an important role in mediating cellular immune responses relevant to AIDS pathogenesis. In the latter analyses, cell mediated immune responses in HIV-negative subjects was assessed by delayed type hypersensitivity (DTH) skin test reactivity following challenge with two separate antigens namely keyhole limpet hemocyanin and purified protein derivative, and concordant associations were observed with both antigens ([20]; DTH responses are an *in vivo* parameter of T cell and cell mediated immune responses, and the strength of DTH responses are an independent determinant of AIDS susceptibility [20], [48], [49], [50], [51], [52], [53]). Finally, the finding that copy number variation in *CCL3*-like genes is also present in chimpanzee [5], macaque [54] and other non-human primate species (our unpublished observations) suggests an evolutionarily conserved host defense function for *CCL3L*-like genes in humans and non-human primates.

To evaluate the genotype-phenotype associations for the combined effects of variations in *CCL3L1* and *CCR5*, we assigned subjects to low, moderate and high *CCL3L1-CCR5* genetic risk groups (GRGs), defined according to different combinations of specific *CCR5* polymorphisms and *CCL3L1* gene copy number [5], [20]. A low, moderate and high *CCL3L1-CCR5* GRG status was associated with a step-wise increase in susceptibility to depletion of CD4^+^ T cells during HIV-1 infection, as well as impaired CD4^+^ T cell recovery during HAART [20], [23]. The association between *CCL3L1*-*CCR5* GRG status and CD4^+^ T cell loss and/or AIDS susceptibility was observed in two separate groups of subjects who were followed from the early stages of their infection within the context of a natural history cohort as well as in a separate cohort of subjects who were recruited during acute/early infection [5], [20], [23]. Furthermore, we observed an enrichment of protective *CCL3L1-CCR5* genotypes in subjects from a natural HIV-1 history cohort who were AIDS-free for more than 10 years, as well as in individuals from two different cohorts who were categorized as spontaneous HIV-1 controllers, i.e., elite or viremic controllers [20], [55], further underscoring that these genetic factors might play an important role in restricting disease progression. The association between *CCL3L1-CCR5* GRG status and immune recovery was assessed in subjects who received HAART during chronic infection using two separate phenotypic endpoints: CD4^+^ T cell count [23] and function [20], assessed by DTH skin test responses. Concordant associations between *CCL3L1-CCR5* genotype and recovery of CD4^+^ T cell counts were also observed in subjects who received HAART during acute/early infection [23].

Collectively, these findings suggested that *CCL3L1-CCR5 g*enetic markers may have dual prognostic capacities, i.e., for both CD4^+^ T cell loss and recovery. A possible role for these genetic markers in evaluation of AIDS risk was underscored by the observation that *CCL3L1-CCR5* GRG status predicted HIV disease course independent of the viral load and CD4^+^ T cell count as well as other explanatory variables that were in themselves independent markers of disease progression (e.g., percent, nadir and cumulative CD4 cell counts, and DTH skin test reactivity) [20]. Notably, the latter associations were observed in two separate and large groups of subjects who within the context of a natural HIV-1 history cohort were followed prospectively from the early stages of their infection [20]. These findings suggested that there is a component of AIDS risk that is genetically-defined by *CCL3L1-CCR5* genetic variations which may not be captured fully by the laboratory markers. The consistency of the disease-influencing associations for *CCL3L1-CCR5* GRG status that were independent of viral load and CD4^+^ T cell count as well as other explanatory variables in two separate groups of HIV-positive subjects prompted us to examine whether *CCL3L1-CCR5* genetic factors might have utility in the clinical management of HIV-infected subjects.

For *CCL3L1-CCR5* GRG status to have utility in the clinical management of HIV, in addition to an influence of *CCL3L1-CCR5* genotypes on CD4^+^ cell recovery during HAART, we surmised that the following four criteria would have to be met. First, the prognostic strength of the GRGs for rates of disease progression should be comparable to that of the CD4^+^ cell count and plasma HIV viral load, contemporary laboratory markers used to assess AIDS prognosis in HIV-positive patients. Second, GRG status should accurately predict an increased risk of developing or progressing rapidly to AIDS when the laboratory markers may fail to do so. Third, the AIDS risk conveyed by the laboratory and genetic markers together, namely the CD4^+^ T cell count plus viral load plus GRG status, should exceed that conveyed by the two laboratory markers, especially during the early stages of infection when subjects have high CD4^+^ T cell counts. Fourth, GRG status should serve as independent determinants of the time interval from seroconversion to CD4^+^ cell count thresholds that are used to guide initiation of HAART. Here, in a large, well-characterized cohort of HIV-1-infected subjects, we investigated whether *CCL3L1-CCR5* GRG status met each of these criteria. The sum of our findings using different analytical approaches suggests that the *CCL3L1-CCR5* GRG status meet these criteria. These findings have broader translational value as they indicate that AIDS risk is an individual characteristic that might be estimated with an increased level of confidence if one also considers the genetic make-up of the host.

## Methods

### Study cohort

In this study, we used the copy number of *CCL3L1* gene [5] and *CCR5* genotype data [5], [18] from a cohort of adult HIV^+^ subjects followed at the Wilford Hall United States Air Force Medical Center (WHMC), San Antonio, TX. This prospective observational cohort is a component of the United States Military's Tri-Service AIDS Clinical Consortium (TACC) Natural History Study [20], [23]. The characteristics of the study subjects (n = 1,132) have been described extensively previously [5], [18], [56], [57] and are summarized in Table S1. The nature of the WHMC HIV^+^ cohort is such that both the seroconverting and seroprevalent groups of patients were followed prospectively from the time of their diagnosis during the early stages of their disease, albeit the approximate time of seroconversion was estimable only in the former group. However, consistent with this recruitment pattern during the early stages of their disease we found that the genotype-phenotype associations detected in these two groups of patients were very similar [20]. For this reason, in this study relevant findings detected in the seroconverters (n = 515) were validated in the seroprevalent (n = 617) component of the cohort. Forty percent of the entire cohort progressed to AIDS (1987 criteria), and 39% died during the study period that ended December 31, 1999. Thus, this cohort was well suited to evaluate our study questions/criteria because all subjects were prospectively followed from the early stages of their infection, and factors that may confound genotype-phenotype studies (e.g., unequal access to medical care and antiretroviral therapy, and loss to follow-up) were not present [5], [20], [23]. The voluntary, fully informed consent of the subjects used in this research was obtained as required by Air Force Regulation 169-9 and additional approval from the Institutional Review Board (IRB) of WHMC and the University of Texas Health Science Center, San Antonio, TX.

### *CCL3L1-CCR5* Genetic Risk Groups

The copy number of *CCL3L1* [5] and *CCR5* haplotype pairs were categorized into three risk categories, and we labeled a patients' *CCL3L1-CCR5* genotype as a low, moderate or a high GRG. The prevalence of the low (*CCL3L1^high^CCR5^non-det^*), moderate (*CCL3L1^high^CCR5^det^* and *CCL3L1^low^CCR5^non-det^*) and high (*CCL3L1^low^CCR5^det^*) GRG in the WHMC cohort is 50, 42, and 8%, respectively [5], [20]. In some analyses, we combined the moderate and high GRG into a single category. Of the 1,132 study subjects, *CCL3L1-CCR5* genotype data for this study was available from 1,103 HIV^+^ subjects and they were 624, 410 and 69 European-, African- and Hispanic-Americans, respectively [5].

### Clinical outcomes and laboratory parameters

The outcomes analyzed were risk of development of AIDS, and rate of progression to AIDS as well as to CD4 cell count thresholds used to guide initiation of HAART [6], [7], [8], [9]. The 1987 Centers for Disease Control (CDC) criteria for AIDS were used in these studies. The laboratory variables studied were baseline CD4 cell counts, steady-state viral load, and DTH skin test responses. DTH responses reflects T cell function *in vivo* (e.g. IL-2 production) and is an independent predictor of disease outcome [20], [50], [51], [52]. Extensive details regarding the DTH testing are provided elsewhere [20], [52].

As described previously [20], [58], we accounted for possible receipt of anti-retroviral therapy (ART) by using the calendar year of membership in the cohort as a proxy for receipt of mono/dual therapy (1990–1996) and HAART (1996-onwards). All subjects who were recruited in the therapy eras were pooled into a single group [20]. The therapy eras and the number of subjects with cohort membership during each era are as described previously [20]. Predictably, membership of a subject to the era in which HAART was available was associated with a significantly reduced risk of progressing to AIDS [20].

### Statistical methods

To address our study questions/criteria, we used the following statistical approaches.

First, to determine whether the *CCL3L1-CCR5* GRGs conveyed a reduced or increased AIDS risk in settings when the laboratory markers would have suggested an opposite risk, we employed the likelihood ratio (LR) statistic. We used this statistic as it more directly describes the effect of a test result (in this case GRG status) on the odds of disease [59]. LR estimates for the possibility of developing AIDS in the future based on the stratum of the laboratory markers as well as for the low, moderate and high GRGs. The stratum for the baseline CD4 cell counts were <350, 350–699, or ≥700 cells/mm^3^, and for the viral load they were < or ≥55,000 copies/ml. These cut-offs were based on published guidelines for assessing AIDS risk that were prevalent during the time these analyses were being conducted. LR was estimated as the ratio of the incidence of AIDS in subjects with a given GRG, CD4 or viral load stratum (i.e., post-test probability of developing AIDS in the study cohort based on the laboratory or genetic markers) and the overall incidence of AIDS in the entire cohort (i.e., pre-test probability of AIDS in the study cohort before accounting for the laboratory or genetic markers). Thus, the LR represents an altered risk in the possibility of developing AIDS in the future based on a subjects' GRG and specific stratum of CD4 or viral load (or the indicated combinations thereof) divided by the overall likelihood of developing AIDS in the HIV^+^ subjects in the WHMC cohort during the study period.

Second, in addition to the LR, we also estimated the pre- and post-test probabilities of developing AIDS during the study period. The LR and probabilities of developing AIDS during the study period were assessed at two levels: we initially calculated LRs and the pretest probability for the laboratory and genetic markers separately (unstratified analysis), and then in the different CD4 and viral load strata in subjects with specific GRGs (stratified analyses).

Third, using a Poisson regression model, we estimated the annual incidence of AIDS in the seroconverting component of the WHMC cohort according to a subjects' laboratory and genetic markers.

Fourth, we derived an estimate of the number of subjects one would need to treat to prevent the occurrence of one case of AIDS (that is, the number-needed-to-treat, NNT). For this, we applied separate Poisson regression models to data from seroconverters who were recruited in eras when antiretroviral therapy (ART) was available (therapy era) or was not available (no therapy era). From these results, the NNT was calculated as follows: If I_n_ and I_t_ represent the estimated incidence of AIDS in the no therapy era (n) and therapy (t) era from the Poisson regression models, respectively, then NNT = 1/(I_n_−I_t_). The estimate of NNT obtained was then rounded to the nearest integer.

Fifth, we used two distinct yet complementary approaches to assess whether the GRGs provided additive value for prognostication of AIDS over and above that provided by the CD4 cell count and viral load. In the first approach, we used a risk scoring system based on cut-offs for CD4 cell counts and the viral load, and conducted survival analyses for time to AIDS. The cut-offs used were based on CD4 cell count and viral load thresholds at which one might consider initiation of therapy. In the risk scoring systems, the cut-offs for CD4 cell counts were at 200, 350 and 450 cells/mm^3^ and for the viral load it was at 55,000 copies/ml; a CD4 cell count of 450 cells/mm^3^ was used as a threshold to determine the relative prognostic value of the laboratory and genetic markers during the early stages of the infection and was an arbitrary cut-off used to reflect early stage disease. Kaplan Meier (KM) survival curves were constructed to graphically illustrate the rate of progression to AIDS, and the log-rank test was used for between-group analysis. We used Cox proportional hazards models to estimate the relative hazards (with 95% confidence intervals (CI)) after testing for the assumption of proportional hazards by plotting the Schoenfeld residuals. Schoenfeld residuals were calculated for each Cox proportional hazards model studied by using the Breslow-Peto approach. Model fit was assessed using the likelihood ratio chi-square (LR χ^2^) and Akaike information criterion (AIC). The critical χ^2^ was estimated by dividing the logrank χ^2^ by its degrees of freedom. The risk-scoring systems were first applied to the seroconverting component of the cohort and then for purposes of replication, we also estimated the LR χ^2^ and AIC estimates in the seroprevalent group of subjects in the WHMC cohort. In the second risk-scoring approach, we used the classification and regression trees (CART) that are commonly used as a method of deductive reasoning for the purposes of data mining and extracting relationships among the predictor variables [60], [61], [62], [63], [64]. The advantage of the CART approach is that the algorithm generates a decision-tree that is free of any preconceived bias with respect to predefined cut-offs for viral load and CD4 cell counts at which ART might be initiated. The decision tree generated by CART for risk of developing AIDS was then used to determine whether it also had applicability for stratifying the rate of progression to AIDS. Extensive details of the model are provided in Section 1 of the Text S1 and Figure S1.

Finally, KM survival curves were used to determine time-from-seroconversion to the CD4 cell count thresholds that might be used to decide when to initiate HAART. We used the thresholds of 200 and 350 as well as 450 cells/mm^3^. We used Stata 7.0 (Stata Corp., College Station, TX) software for all statistical analyses and the program DTREG (Brentwood, TN) for generation of the classification trees.

## Results

The four criteria that might provide a framework for considering *CCL3L1-CCR5* GRG status in the clinical management of HIV-infected subjects were addressed as follows.

### Criterion one

To determine whether the genetic and laboratory markers had comparable prognostic capacities, we first conducted unstratified analyses in which we computed the probabilities and likelihood ratio (LR) of developing AIDS based on a subject's baseline CD4 or steady-state viral load stratum before accounting for that person's GRG. Similar estimates for the GRGs were computed before accounting for a subject's CD4 or viral load (Table 1). The pretest probability of developing AIDS in the entire cohort and the seroconverting component of the cohort in whom *CCL3L1-CCR5* genotype was determined was 39% and 27%, respectively. However, the post-test probabilities of developing AIDS differed according to a person's GRG or CD4 cell count (Table 1). Notably, in the overall cohort, the posttest probabilities (parenthesis) for a low (32%), moderate (43%), and high (66%) GRG were similar to those for a baseline CD4 cell count of ≥700 (26%), 350–699 (40%) and <350 (61%) cells/mm^3^ (Table 1; values in parentheses denote the posttest probabilities, and for GRG status they are the estimates shown in the bottom-most row of Table 1, whereas for the CD4 cell count they are the estimates shown under the column “unstratified by GRG”).

**Table 1:** The likelihood ratio (LR) and pre- and post-test probability of developing AIDS according to the laboratory markers and *CCL3L1-CCR5* GRGs.

		Unstratified by GRG	Stratified by GRG		
			Low GRG	Moderate GRG	High GRG
CD4 or Viral load strata	N	LR (95% CI) [pre-test, post-test]	LR (95% CI) [pre-test, post-test]	LR (95% CI) [pre-test, post-test]	LR (95% CI) [pre-test, post-test]
CD4+ T cells (cells/mm^3^)*					
<350	186	2.44 (1.87–3.19) [39%, 61%]	0.69 (0.51–0.93) [61%, 52%]	1.01 (0.71–1.45) [61%, 62%]	13.3 (1.83–96.3) [61%, 95%]
≥350	916	0.83 (0.78–0.88) [39%, 35%]	0.74 (0.64–0.86) [35%, 28%]	1.22 (1.04–1.42) [35%, 40%]	2.34 (1.45–3.77) [35%, 56%]
350–699	566	1.04 (0.92–1.16) [39%, 40%]	0.78 (0.65–0.94) [40%, 34%]	1.12 (0.94–1.35) [40%, 43%]	2.23 (1.24–4.02) [40%, 60%]
≥700	350	0.55 (0.45–0.67) [39%, 26%]	0.70 (0.54–0.91) [26%, 20%]	1.35 (1.03–1.78) [26%, 32%]	2.40 (1.03–5.60) [26%, 46%]
Viral load†					
≥55 k	95	2.22 (1.57–3.13) [27%, 45%]	0.73 (0.44–1.20) [45%, 37%]	1.06 (0.66–1.68) [45%, 46%]	2.33 (0.76–7.20) [45%, 66%]
<55 k	310	0.74 (0.64–0.87) [27%, 22%]	0.69 (0.51–0.93) [22%, 16%]	1.31 (0.96–1.81) [22%, 27%]	3.19 (1.28–7.95) [22%, 47%]
CD4 plus viral load†					
≥350 and <55 k	270	0.64 (0.52–0.79) [27%, 19%]	0.70 (0.50–0.97) [19%, 14%]	1.27 (0.88–1.83) [19%, 23%]	3.28 (1.28–8.39) [19%, 43%]
All subjects*	1103		0.73 (0.64–0.83) [39%, 32%]	1.16 (1.01–1.34) [39%, 43%]	3.02 (1.96–4.64) [39%, 66%]

The unstratified analyses are posttest and LR estimates for the baseline CD4^+^ T cell count and steady-state viral load prior to accounting for a subject's GRG and are in the column denoted as “unstratified by GRG”, whereas similar estimates for the GRGs before accounting for a subject's baseline CD4 cell count and steady-state viral load are in the bottom-most row.

* Data are for the entire cohort.

† Data are for seroconverting subjects and indicates the steady-state viral load in these subjects. k, denotes×10^3^ copies/ml.

The percentages in the brackets [ ] denote the pre-test followed by the post-test probability of developing AIDS.

A LR of >1 or <1 indicates a greater or lower likelihood of developing AIDS, respectively, and we found that the three CD4 and viral load strata as well as a low, moderate and high GRG status were each associated with predictable LRs (Table 1). For example, LRs for a low, moderate and high GRG were 0.73, 1.16, and 3.02, respectively (Table 1, bottom-most row). The LR estimates for a low, moderate and high GRG were quantitatively very similar to the LRs (estimates in parenthesis) for a baseline CD4 cell count of ≥700 (0.55), 350–699 (1.04) and <350 (2.44) cells/mm^3^ (the latter estimates are in the column denoted as “unstratified by GRG” in Table 1). Similarly, the LR (parenthesis) for a low (0.73) and high (3.02) GRG were similar to those for a viral load of <55,000 (0.74) and ≥55,000 (2.22) copies/ml, respectively (the latter estimates are in the column “unstratified by GRG” in Table 1). Thus, the results of these unstratified analyses indicated that the posttest probabilities and LR estimates of developing AIDS associated with a low, moderate or high GRG were remarkably similar to those conveyed by strata of laboratory markers that are known to prognosticate a low, moderate or high risk of AIDS.

### Criterion two

However, because a low, moderate and high GRG status associate with different degrees of CD4 cell loss and viral replication, an argument could be made that the GRGs do not add any additional prognostic information than that conveyed by the laboratory markers. Thus, we sought to determine whether GRG status predicted risk of AIDS when the CD4 and viral load may not. For this, we conducted stratified analyses in which we computed the LRs for the CD4 and viral load strata but after accounting for a subject's GRG (Table 1, columns under “stratified by GRG”). Three examples are used to illustrate that GRG staus accurately conveyed a reduced or increased AIDS risk, even in instances when the laboratory markers in themselves would have predicted a contrary risk of developing AIDS (Table 1). First, the LR and posttest probability values for a CD4 cell count of <350 cells/mm^3^ were 2.44 and 61%, respectively, but for a low, moderate, or high GRG in this CD4 strata they were 0.69 and 52%, 1.01 and 62%, and 13.3 and 95%, respectively (Table 1, top row). Hence, among subjects in whom a low CD4 count predicted an increased likelihood of developing AIDS, a low GRG tracked a subset of these subjects who have a lower risk of developing AIDS, whereas a high GRG identified a subset of individuals in this vulnerable CD4 cell stratum of <350 cells/mm^3^ who have an even higher risk of developing AIDS.

Second, the LR and posttest probability values for a CD4 cell count of ≥700 cells/mm^3^ were 0.55 and 26%, respectively; but for a low, moderate, or high GRG within this CD4 stratum they were 0.70 and 20%, 1.35 and 32%, and 2.40 and 46%, respectively (Table 1). In this instance, among subjects in whom a high CD4 count would have predicted a lower likelihood of developing AIDS, a high and moderate GRG identified subsets of subjects with increased likelihoods of developing AIDS. Third, in the seroconverting subjects, the LR and posttest probability values for a CD4 cell count of ≥350 cells/mm^3^ and a viral load of <55,000 copies/ml were 0.64 and 19%; these values were 0.70 and 14%, 1.27 and 23%, and 3.28 and 43% for those assigned to a low, moderate, or high GRG, respectively (Table 1, estimates shown under row entitled “CD4 plus viral load”). Results of these analyses indicated that knowledge of the CCL3L1-CCR5 GRG status may improve the ability to predict AIDS risk, especially by identifying those subjects whose CD4 and viral load parameters may incorrectly predict a contrary risk of developing AIDS.

### Criterion three: approach #1 – risk-scoring systems

To determine whether GRG status added to the prognostic capacity of the CD4 cell count and viral load in predicting the risk of developing AIDS in seroconverters, the CD4 cell count and viral load were stratified as shown in Table 2. Predictably, the incidence of AIDS was much greater in those subjects whose membership in the cohort did not coincide with the therapy era (range: 4.3 to 52.3) than in those whose membership in the cohort coincided with the therapy era (range: 1.1 to 19.6; Table 2). However, irrespective of therapy eras, within each CD4/viral load stratum, low, moderate and high GRG status conveyed a step-wise increase in AIDS risk, which was also evident among subjects with the lowest risk CD4 cell count (≥700 cells/mm^3^) and viral load (<20,000 copies/ml) profile (Table 2). Based on these analyses, we determined the number of patients that one would need to treat with HAART to prevent one case of AIDS if the GRG status of the patient were accounted for (Table 2). These analyses suggested that in each CD4/viral load stratum, compared to those with a high GRG, twice the number of subjects with a low GRG would need to be treated to prevent one case of AIDS (Table 2).

**Table 2:** Annual incidence of AIDS (%) and numbers needed-to-treat according to baseline CD4^+^ T cell count, the steady-state viral load and *CCL3L1-CCR5* GRG, based on Poisson regression model.

GRG	CD4≥700			CD4 350–699			CD4<350		
	Viral load			Viral load			Viral load		
	<20 k	20-<55 k	≥55 k	<20 k	20-<55 k	≥55 k	<20 k	20-<55 k	≥55 k
Annual Incidence of AIDS									
All patients (N = 402)									
Low	1.6	3.3	4.6	2.0	4.2	5.9	3.4	7.1	9.9
Moderate	2.3	4.7	6.6	2.9	6.0	8.3	4.9	10.1	14.1
High	4.8	9.8	13.7	6.0	12.4	17.3	10.2	20.8	29.1
Patients not in HAART era (N = 97)									
Low	4.3	7.3	11.9	4.8	8.1	13.3	8.8	15.0	24.5
Moderate	5.3	9.1	14.9	5.9	10.2	16.6	11.0	18.7	30.6
High	9.1	15.5	25.4	10.1	17.3	28.4	18.7	31.9	52.3
Patients in HAART era (N = 305)									
Low	1.1	2.1	2.4	1.5	2.9	3.2	3.6	6.9	7.7
Moderate	1.6	3.0	3.4	2.2	4.1	4.6	5.2	9.8	10.9
High	2.8	5.4	6.0	3.9	7.3	8.2	9.2	17.5	19.6
Numbers needed-to-treat									
Low	32	19	10	31	10	10	19	12	6
Moderate	27	16	9	26	16	8	17	11	5
High	16	10	5	16	10	5	11	7	3

Results are from the seroconverting component of the cohort. CD4 cell count (baseline) is in cells/mm^3^. k, denotes×10^3^ copies/ml. The assignment of subjects whose membership in the WHMC cohort fell in the eras in which HAART was available or not, and the impact of the HAART era on rates of disease progression are as described previously [20].

**Figure 1:**
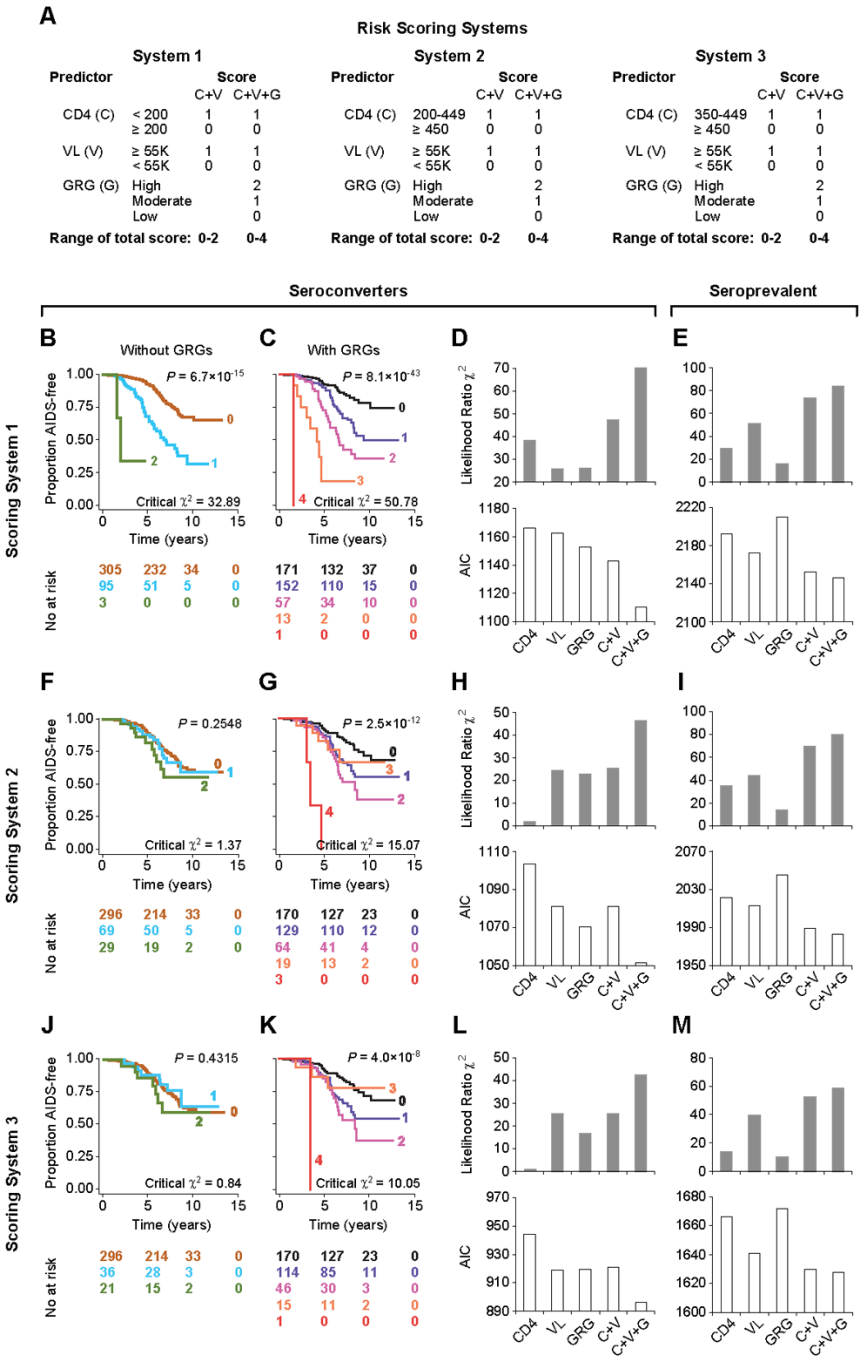
Prognostic performance of the *CCL3L1-CCR5* GRGs in risk-scoring systems. Panel A shows the three risk-scoring systems based on baseline CD4^+^ T cell counts (C), viral load (V) and GRGs (G) in the seroconverting and separately in the seroprevalent component of the WHMC HIV^+^ cohort. Panels B–C, F–G and J–K depict Kaplan Meier (KM) plots for progression to AIDS (1987 criteria) before and after accounting for the GRGs in the risk-scoring systems indicated on the left axis. The critical ratio χ^2^ values are indicated within the KM plots. Panels D, H and L depict likelihood ratio χ^2^ and AIC estimates in seroconverters, and panels E, I and M depict the same estimates for the seroprevalent subjects.

The preceding findings underscored that over and above the AIDS risk conveyed by the CD4 cell count and viral load, *CCL3L1-CCR5* GRG status may serve as a unique parameter to identify HIV-infected individuals who are at an increased risk of developing AIDS. To investigate further this possibility, we considered whether GRGs and laboratory markers can be combined in a clinically meaningful way to enhance AIDS prognostication. As risk scoring systems have an inherent ease of applicability in a clinical setting, we used this as an approach to evaluate whether the prognostic value of GRGs might have value in clinical practice. We determined whether a three-pronged risk-scoring system that included the two laboratory markers and GRG status was superior in fractionating the risk of progressing rapidly to AIDS when compared to a two-pronged risk-scoring system that excluded GRG status. The first scoring system stratified the seroconverting component of the cohort into two groups based on whether their baseline CD4 cell count was < or ≥200 cells/mm^3^ (Figure 1A, and B to E); the second scoring system used a CD4 cut-off of 450 cells/mm^3^ but excluded the subjects with <200 CD4-cells/mm^3^ (Figure 1A and F to I); and the third scoring system also used a cut-off of 450 cells/mm^3^ but excluded those with a CD4 cell count of <350 cells/mm^3^ (Figure 1A and J to M). By excluding subjects with CD4 counts of <200 or <350 and by placing the upper CD4 cell count threshold at 450 cells/mm^3^, the second and third risk-scoring systems offered the opportunity to explore whether GRG status can prognosticate AIDS risk during the earlier stages of the disease, i.e., when the baseline CD4 cell count is higher.

In each of the three risk-scoring systems tested (Figure 1, B to D, F to H, and J to L), the prognostic performance of the nested models and survival curves that contained GRG status along with the CD4 and viral load (i.e., C+V+G) were superior to those risk-scoring systems that excluded the GRGs (i.e., C+V). Concordant effects of the GRGs were also found in the seroprevalent component of the cohort (Figure 1, E, I and M). The additive prognostication conveyed by the GRGs was indicated by (i) the Kaplan-Meir (KM) plots in which the disease-modulating effects of the GRGs were accounted for displayed improved stratification for different rates of disease progression than those plots in which only the effects of CD4 cell count and viral load were accounted for; (ii) higher critical χ^2^ values for the Cox models that contained the GRGs (values are in the panels depicting the KM plots; e.g., compare critical χ^2^ of 0.84 and 10.05 in panels J and K in models lacking and containing the GRGs, respectively); and (iii) higher likelihood ratio χ^2^ and lower AIC values in models that included the GRGs (Figure 1, panels D–E, H–I, L–M).

The KM plots showed that the CD4 cell count and viral load provided the most prognostication only in the first scoring system, i.e., when subjects with a CD4 of <200 cells/mm^3^ were included in the model (Figure 1, compare panel B versus panels F and J). Supporting this, the prognostic capacity of the CD4 cell count as reflected by the estimates for the likelihood ratio χ^2^ and AIC were most apparent in subjects with a CD4 count of <200 cells/mm^3^ (Figure 1, D and E), but negligible in the other two scoring systems in which the CD4 cell counts were >200 cells/mm^3^ (Figure 1, H, I, L and M). Notably, in the second and third risk-scoring systems, the values for the likelihood ratio χ^2^ and AIC were similar for the viral load and the GRGs (Figure 1, H, I, L and M), suggesting that the prognostic information conveyed by the viral load and GRG status in these two risk-scoring systems were comparable.

### Criterion three: approach #2 – CART analysis

**Figure 2:**
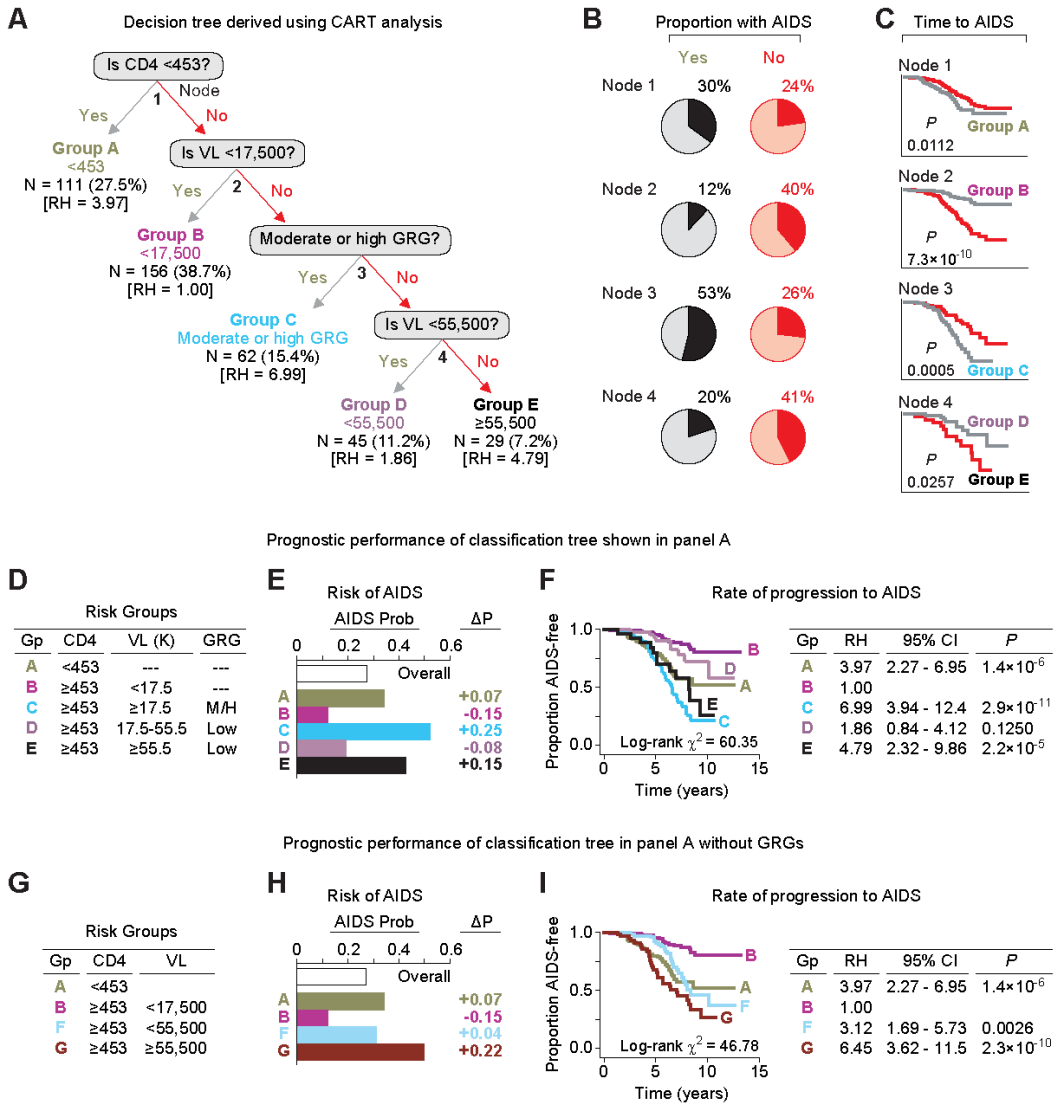
Classification trees and their application in the seroconverting component of the WHMC HIV^+^ cohort. (A) A binary decision tree derived by CART analysis for the risk of developing AIDS (1987 criteria) based on baseline CD4^+^ T cell counts, viral load (VL) and GRG status in the seroconverting component of the WHMC HIV^+^ cohort. The analysis identified five exclusive groups designated as Groups A to E. The tree shows that the proximal split was based on the CD4 cell count, and the CART analysis generated the cut-off to be 453 cells/mm^3^. The next split was based on a viral load of 17,500 copies/ml. The third split was based on GRG status, followed by another split at a viral load of 55,500 copies/ml. The five groups generated are color-coded and the number of subjects in each of these groups is shown along with their proportion in the study group. The values in brackets ([ ]) indicate the relative hazard estimates corresponding to each of these groups as shown in Figure 2F. (B) Pie charts depicting the proportion of subjects in each of the splits who did or did not develop AIDS (1987 criteria). Within each pie-chart, the dark pie-slice represents the proportion of subjects who developed AIDS. Yes and no refers to whether a subject does or does not categorize, respectively, to the indicated node. (C) KM plots for the rate of progression to AIDS from time of seroconversion based on the split at each corresponding node in the decision tree shown in panel A. The significance values shown below each KM plot were estimated using the logrank test. (D–F) Association between the five risk groups (panel D) generated by CART and the risk (panel E) and rate of developing AIDS (panel F). Panel D defines the risk groups based on the baseline CD4 (cells/mm^3^), steady state viral load (k, ×1,000 copies/ml), and GRG status (M/H, moderate or high GRG; Low, low GRG). Gp, group. Panel E shows the probability (Prob) of developing AIDS within each risk group generated by CART analysis. “Overall” refers to the probability of developing AIDS in the seroconverting component of the cohort without accounting for the laboratory or genetic markers. ΔP, change in probability from the overall probability. Panel F shows the KM plots for rate of progression to AIDS for the five groups of subjects identified by CART. The table to the right shows the relative hazards (RH) corresponding to these five groups estimated by using Cox proportional hazards models. In these analyses, the reference category (RH = 1) is group B, which denotes subjects that have a CD4 of ≥453 cells/mm^3^ and a viral load of <17,500 copies/ml. The results show that relative to this reference category, groups A, C and E are associated with a significantly increased risk of progressing rapidly to AIDS. (G–I) Similar analyses to those shown in panels D to F but using risk groups in which the GRGs are not included as prognosticators. In these analyses, the risk groups A and B shown in panel A and panel D were used along with two new groups designated as group F and group G. The latter two groups were derived from the Groups C to E shown in panel D by not accounting for the GRG status and dichotomizing the cohort further based on a viral load cut-off of 55,500 copies/ml. Reference category (RH = 1) is group B. In panels E and H, prob refers to probability.

The predefined cut-offs for CD4 cell counts and the viral load in a risk-scoring system is useful because they reflect thresholds currently used to decide the initiation of HAART (e.g., CD4≤200 or 350 cells/mm^3^). However, the use of these cut-offs has the limitation of potentially biasing the risk-scoring system towards an improved discrimination of AIDS risk. For this reason, we used an additional approach to address criterion 3 and assessed the role of the GRGs in AIDS prognostication by using a classification and regression tree (CART) approach (Figure 2A and Figure S1). Based on the differential risk of developing AIDS, CART analysis identified a decision tree defined by four nodal points that stratified the cohort into five groups, denoted as groups A to E (Figure 2A and Figure S1A). Interestingly, the most proximal node defined by CART analysis was a CD4-cell count of 453 cells/mm^3^, which was similar to the CD4 cell count threshold we used in the aforementioned analyses in risk-scoring systems (i.e., 450 cells/mm^3^, Figure 1A). Additional nodes included the GRG and steady-state viral load of these subjects (Figure 2A and Figure S1A). In this instance, the nodal split for viral load defined by CART analysis was at 55,500 copies/ml, and this was similar to the cut-off for viral load that we used in the analyses shown above (i.e., 55,000 copies/ml; Tables 1 and 2 and Figure 1). The manner in which these splits stratified subjects with variable risks of AIDS development at each node is shown (Figure 2A and 2B).

To assess if the nodal splits derived based on differential risk of AIDS development also conveyed differential rates of disease progression, we plotted KM plots for each binary split shown in the decision tree (Figure 2C). We observed that each nodal split was also associated with statistically significant differences in the rates of progression to AIDS (Figure 2C).

The five risk groups (Figure 2D) defined by the CART analysis displayed strikingly different risks of future development of AIDS (Figure 2E) as well as rates of disease progression (Figure 2F). Compared to the pretest probability of developing AIDS in the overall seroconverting component of the cohort (27%), Group C, which is defined by individuals with a CD4 count of ≥453 cells/mm^3^, a viral load of ≥17,500 copies/ml, and moderate or high GRG status, was associated with a 25% excess probability of developing AIDS. By contrast, group B (subjects with a relatively high baseline CD4 count (≥453 cells/mm^3^) and low steady-state viral load (<17,500 copies/ml)) was associated with a 15% reduced probability of developing AIDS in the future (Figure 2F). Cox proportional hazards modeling demonstrated that Group C was associated with the fastest rate of progression to AIDS, whereas group B (subjects with a CD4 cell count of <453453 cells/mm^3^) was associated the slowest rate of disease progression (Figure 2F).

To validate that GRG status provided additional predictive value in the decision tree, we excluded GRG status from the analysis, and determined the risk and rate of progressing to AIDS for the four risk groups defined by the CD4 cell count and viral load cut-offs generated by CART (Figure 2G). A comparison of the identical analyses for the five (Figure 2D) versus four (Figure 2G) risk groups revealed that inclusion of GRG status within the stratification schema provided more a refined risk-profile than a stratification schema that excluded GRG status (compare Figure 2, E versus H and F versus I). The overall discriminatory power for prognosticating subjects with differential rates of progression to AIDS was improved by ∼29% when the GRGs were included for prognostication (*P* = 0.0002; also compare the log-rank χ^2^ values in the KM plots in Figure 2F (χ^2^ = 60.35) and 2I (χ^2^ = 46.78), showing higher values when GRG status is included in the model).

The analyses in Figure 2 are for the seroconverting component of the WHMC HIV^+^ cohort, and we replicated these observations in the seroprevalent component of this cohort (Figure S2). Hence, the findings of two distinct risk-stratification approaches (Figures 1 and 2, and Figure S2) in two large and separate groups of HIV^+^ subjects demonstrated that the GRGs improve the ability to capture the wide spectrum of AIDS risk. Remarkably, the CART approach also showed that the *CCL3L1-CCR5* GRGs assist in AIDS prognosis mainly during early-stage disease, such as when baseline CD4 cell counts are greater than 453 cells/mm^3^, a CD4 cell count threshold that is well above the point at which ART is currently recommended.

### Criterion four

**Figure 3:**
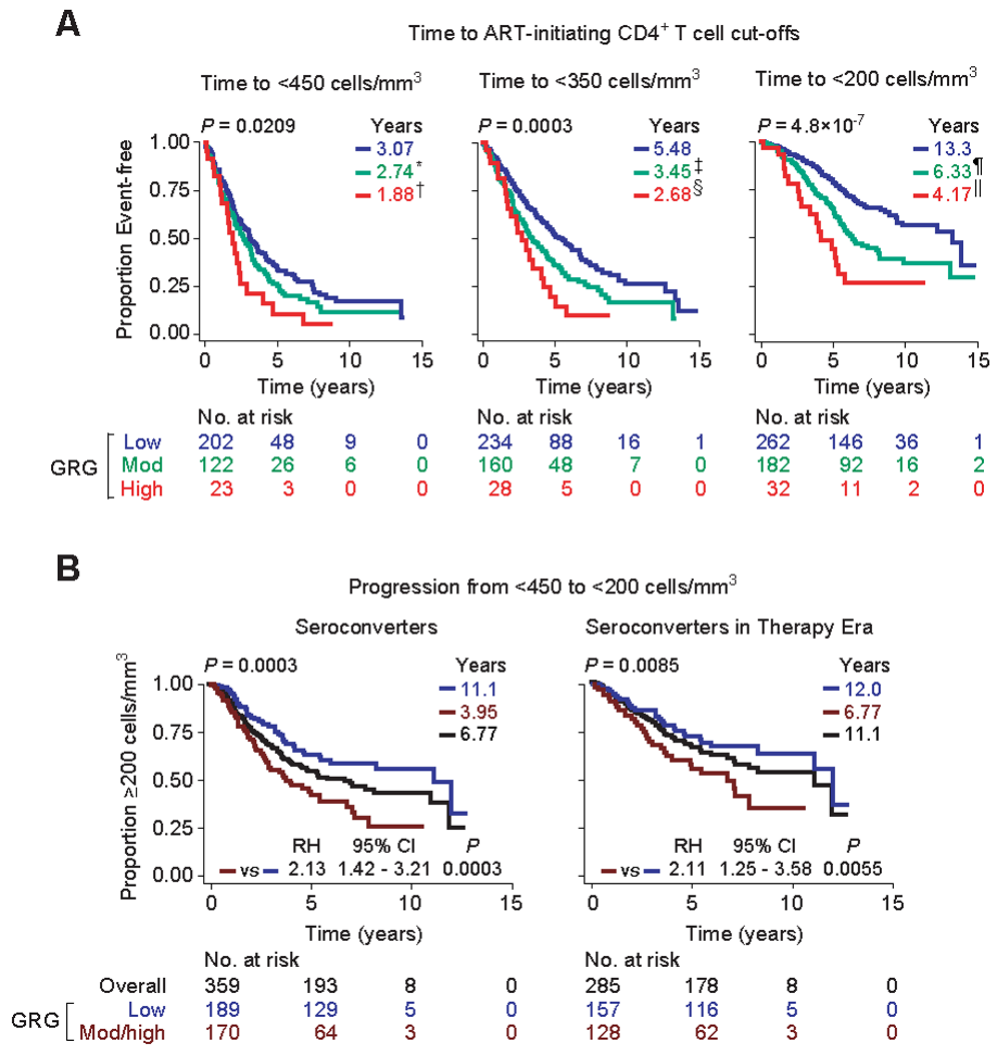
*CCL3L1-CCR5* GRGs influence median time-from-seroconversion to CD4 cell count thresholds that might be used to guide initiation of HAART, and time from a high to a low CD4 cell count. In Panel A, KM plots show the time-from-seroconversion to arrival at <450 (left), <350 (center) or <200 (right) CD-cells/mm^3^. In Panel B, the KM plots are for progression from <450 to <200 CD4-cells/mm^3^ in all seroconverters (left) or seroconverters recruited and followed during the years 1990 to 1999, a time-period in which antiretroviral therapy was available (right). The color codes for the KM plots in panel B are: blue, low GRG; brown, moderate and high GRGs combined into a single category; and black, all subjects. Overall *P* values are at the top of each plot. Color-coded numbers at the upper right of the KM plots represent the median time-to-event, that is, from seroconversion to the indicated CD4 cell count. In panel A, *P* values for differences in median time-to-event relative to those with a low GRG were: *, 0.1131; †, 0.0089; ‡, 0.0030; §, 0.0005; ¶, 0.0001; and ∥, 9.6×10^−7^. RH, relative hazard; CI, confidence interval. *P* values in Panel B are adjusted for the steady-state viral load, baseline CD4 and best DTH response recorded during disease course, and subjects with a moderate or high GRG were combined into a single category.

The aforementioned findings indicated that along with the CD4 cell count and viral load, the GRGs provided additive prognostication during the early stages of HIV infection, implying that the GRGs might prognosticate the time-interval from seroconversion to arrival at CD4 cell count thresholds relevant to commencing therapy. Given the uncertainty surrounding the optimal time to initiate HAART [7] as well as the results of our CART analysis, we chose three CD4 cell count cut-offs to reflect early (450 to approximate the 453 cut-off defined by CART analysis), intermediate (350), and late (200) HAART-initiating CD4 cell count thresholds. These analyses revealed that there was a step-wise increase in the time-interval from seroconversion to arrival at each of these CD4 cell count thresholds in patients with a high, moderate and low GRG (Figure 3A). Thus, it took nearly twice as long for subjects with a low GRG status to arrive at a CD4 cell count threshold of <350 cells/mm^3^ than subjects with a high GRG (5.48 vs 2.68 years; Figure 3A, middle).

Among seroconverting subjects who had <450 CD4 cells/mm^3^, the mean CD4 cell counts did not differ according to a subjects' GRG (mean in different GRGs was 375–383 cells/mm^3^). However, those with a low GRG status took nearly seven years longer to progress from <450 to <200 cells/mm^3^ than all other subjects (Figure 3B). The difference in the rate of progression from <450 to <200 cells/mm^3^ between those who were or were not assigned to a low GRG remained significant even after adjustment for the baseline CD4 cell count and viral load, the best DTH skin test reactivity recorded during disease course, and cohort membership in the calendar years 1990–1999, a time-period during which most of the AIDS events in this cohort occurred and HAART was available (Figure 3B, right).

## Discussion

In this study, we tested the hypothesis that in conjunction with laboratory markers currently used to assess AIDS risk, the complex patterns of genetic variations in the human genome may have utility as tools for improved risk assessment of patients infected with HIV-1. The results of this study conducted in a large, and well-characterized natural history cohort of HIV-infected individuals indicate that in addition to the traditional markers of vulnerability (baseline CD4+ T cell count, viral load and rate of CD4^+^ T cell decline), the inclusion of a measure of genetic risk might offer an adjunctive, and complementary risk-stratification tool that may provide an improved method for identifying persons at high risk for future AIDS related events. These HIV vulnerable individuals may be ideal candidates for preventive AIDS care such as a closer follow-up, and potentially, earlier initiation of HAART. This inference is based on the following four principal findings.

First, the predictive capacities of a low, moderate and high GRG for risk of developing AIDS, as estimated by the likelihood ratio, were equivalent to those of the strata of the laboratory markers that were associated with a low-, moderate- and high-risk of developing AIDS.

Second, although the GRGs and the viral load had similar capacities for risk prognostication, GRG status was not a surrogate marker for the viral load. Instead, GRG status by consistently tracking individuals with reduced or increased likelihoods of developing AIDS provided prognostic information even in those instances when the laboratory markers predicted a contrary likelihood of developing of AIDS. Thus, the GRGs provided prognostic information with three unique characteristics, (i) independence from the AIDS risk conveyed by the viral load and CD4 cell count; (ii) equivalence in magnitude to the laboratory markers; and (iii) ability to correctly predict AIDS risk in spite of contrary information imparted in some instances by the viral load and CD4 cell count.

Third, the results from several complementary but distinct approaches demonstrated that the genetic and laboratory markers provide additive prognostic information that gauges the continuum of the risk of AIDS and disease progression more accurately than either marker alone. Importantly, the additive predictive capacity of the GRGs was evident during the early stages of the infection such as when the CD4 cell count was greater than 450 cells/mm^3^. It is especially noteworthy that in subjects with CD4≥453 cells/mm^3^ and viral load of ≥17,500 copies/ml (∼15% of the study cohort), a moderate or high GRG status was associated with a nearly seven-fold increased risk of progressing rapidly to AIDS.

Fourth, *CCL3L1-CCR5* GRG status served as an independent determinant of how quickly HIV-infected subjects progressed to thresholds of CD4 cell counts that are currently used to decide when to initiate HAART. As a general rule, subjects with a moderate or high GRG progressed to these HAART-initiating CD4 cell count thresholds two to three times faster than those with a low GRG.

Genetic risk stratification of HIV-infected patients is especially attractive for three reasons. First, *CCL3L1-CCR5* genotypes are not only independent determinants of disease, but more importantly, they are also capturing different aspects of AIDS risk than the traditional components of AIDS risk reflected in the laboratory markers currently used to assess disease status. Second, the time-insensitive nature of the genetic markers and the capacity of the GRGs to assess genetically-defined AIDS risk, especially during the proximal stages of infection, is appealing because there is substantial data suggesting that it is the magnitude of early immune damage that dictates, in part, steady-state viral load, initial CD4^+^ loss, and subsequently, pace of HIV disease course [20], [26], [65], [66], [67]. Third, the prevalence of the genetic factors that we have identified in a U.S.-based cohort of HIV-positive individuals (prevalence of the low, moderate and high GRGs is ∼50, 42 and 8%, respectively) is large enough to be of clinical relevance as these genetic factors can provide dual prognostication: risk of AIDS and disease progression, and recovery of CD4 cell numbers [23] and function [20] during HAART.

The robustness of the associations detected for *CCL3L1-CCR5* GRG status is suggested by several observations. First, concordant results were obtained when using different analytical approaches and when using a stringent clinical phenotypic endpoint namely AIDS (1987 CDC criteria). The finding that the copy number of *CCL3L1* and *CCR5* genotypes together provided prognostication during the early stages of the disease (CD4 cell counts of >450 cells/mm^3^) was detected using two separate approaches: risk-scoring systems (Figure 1) and CART (Figure 2). Additionally, consistent results for the latter two analyses were observed in two separate groups of subjects from the same cohort (Table S1), both of whom were followed prospectively from the early stages of their infection [20]. Furthermore, concordant results were obtained when the survival curves for the association between *CCL3L1-CCR5* GRGs were computed from either time-of-seroconversion to different CD4 cell count thresholds (Figure 3A) or from one CD4 cell count threshold to another (i.e., progression from <450 to <200 CD4-cells/mm^3^; Figure 3B), and the latter associations remained consistent after adjustment for other explanatory variables known to influence disease progression rates.

One needs to be cognizant of an important point with regards to the interpretation of our results. The genetic variations in *CCR5* that affect HIV disease course differ in European and African Americans [18], [56], and the average copy number of *CCL3L1* varies among populations [5]. Thus, we caution that as the genetic information was derived from European and African American HIV-positive subjects and because host-virus interactions are likely to be highly population-specific, the composition of the GRGs might vary depending on the study population. Despite this caveat, it is noteworthy that the impact of *CCL3L1* on HIV-AIDS susceptibility has been observed in different geographic regions ([5], [43], [44], [45], [46] and unpublished observations), and that we and others have found reproducible effects of specific *CCR5* haplotype pairs on disease progression rates (e.g. detrimental and beneficial impact of the *CCR5 HHE/HHE* and HHC/HHG*2 genotypes, respectively [15], [17], [18], [19], [39]). Illustrating a basis for the consistency of the disease-influencing associations, these *CCR5* haplotypes are also associated with increased (*CCR5-HHE*) or decreased (*HHC* or HHG*2) gene/protein expression of CCR5 [39], [68], [69], [70], [71], and of note, CCR5 density is in itself a determinant of HIV susceptibility [69], [72], viral load [73], disease progression rates [74], and CD4 recovery during HAART [75], [76].

These findings also have value for improving our understanding of the factors that influence HIV-AIDS pathogenesis and for the interpretation of genotype-phenotype association studies in general. The ability of the GRGs to provide an additive measure of AIDS risk, including in those with high CD4 cell counts, suggests that the impact of these host factors during chronic infection is partly through viral load-independent mechanisms that are likely operative in the proximal end of the causal pathways that affect CD4 cell loss and establishment of the steady-state viral load. This inference is consistent with those derived from the results our previous studies where we suggested that a common *CCL3L1-CCR5* genetic pathway may regulate the balance between pathogenic and reparative processes from the early stages of the disease course [19], [20], [23]. These findings suggest that in addition to their roles in HIV cell entry [1], CCR5 and its ligands may also influence disease pathogenesis by impacting on parameters that are not dependent on viral entry. This is consistent with the observation that CCR5 and its ligands associate with a wide array of immunological responses relevant to HIV-AIDS pathogenesis, including cell-mediated immunity, T cell regeneration, activation-induced cell death, and formation of the immunological synapse [77], [78], [79], [80], [81], [82], [83], [84], [85], [86], [87], [88], [89], [90]. However, it is likely that the relative impact of these *CCL3L1-CCR5*-dependent immune responses on HIV-AIDS pathogenesis may differ depending on disease stage. Furthermore, the influence of *CCL3L1-CCR5* genotypes on HIV-AIDS pathogenesis is evident at time of viral exposure (i.e., HIV acquisition) and soon after seroconversion [5], [20], [23], and as shown herein, these genetic factors provide independent prognostic information from the early stages of the disease. Hence, from an epidemiological standpoint the aforementioned observations suggest that genotype-phenotype associations for *CCL3L1* copy number and *CCR5* genotype may differ significantly depending on the characteristics and disease stage of the subjects evaluated (e.g., those enrolled from the early stages of their infection as part of a natural history cohort versus accrual of subjects during the later stages of disease, or those enrolled as part of a clinical trial, or selection of subjects based on specific clinical or laboratory characteristics).

The notion that a large component of AIDS pathogenesis might be conveyed by parameters that are independent of the viral load is suggested by our previous findings where we found that steady-state viral load and *CCL3L1-CCR5* GRG status each explained ∼9% and ∼6%, respectively, of the variability in rate of progression to AIDS [20]. The studies by Rodriguez et al also underscore the notion that viral load only explains a small fraction of the variability in the rate of decline in CD4^+^ cell counts [91]. Thus, the finding that the *CCL3L1-CCR5* GRGs influence AIDS pathogenesis partly independent of the viral load suggests that strategies to block viral load-independent pathways, such as those linked to the CCL3L1-CCR5 axis, might provide a novel means to curb CD4 cell loss during infection and aid immune reconstitution. Whether this can be accomplished with the current generation of CCR5 blockers needs to be carefully evaluated because studies with a small molecule CCR5 blocker [92] and a CCR5 monoclonal antibody [93] suggest a beneficial effect of blocking CCR5 on CD4^+^ T cell recovery during HAART, in instances when the prevailing viral strain is X4-tropic and independent of viral load suppression, respectively.

In summary, we have conducted proof-of-concept analyses to determine whether genetic information can be used to bring us one step closer towards personalized HIV medicine. Using several different approaches, in this prospective study of HIV-1 infected individuals, the predictive capacity of *CCL3L1-* and *CCR5*-based genetic risk stratification was not only independent of, but more importantly, comparable to the prognostic information provided by currently used predictors of AIDS risk. Thus, additional studies will be required to determine whether in a manner analogous to the use of HIV genotype, when applied judiciously along with knowledge of the clinical and laboratory parameters, host genotypes (e.g., *CCL3L1-CCR5*, *HLA* alleles) that associate with different aspects of HIV disease pathogenesis, independent of CD4 cell count and viral load, might have practical utility in guiding the care of infected individuals. Of broad relevance, using HIV as a model system, we outline a series of analyses that might have application to other diseases when assessing whether genetic information can be used to improve the clinical care of patients afflicted with these diseases.

## Supporting Information

Text S1 Supplemental Online Materials - Text
(0.07 MB DOC)

Figure S1 Classification trees and their clinical application in the HIV+ WHMC cohort.
(0.35 MB TIF)

Figure S2 Replication of results of CART analysis in the seroprevalent component of the WHMC HIV+ cohort.
(0.31 MB TIF)

Table S1 (0.04 MB DOC)
