# Supplementary material for: CCL3L1-CCR5 Genotype Improves the Assessment of AIDS Risk in HIV-1-Infected Individuals
Source: PLoS One. 2008 Sep 8;3(9):e3165. doi: 10.1371/journal.pone.0003165 (PMC2522281; doi:10.1371/journal.pone.0003165)
Supplement: Text S1 — Supplemental Online Materials - Text (0.07 MB DOC) [file pone.0003165.s001.doc]

**Supplementary Material for**

***CCL3L1-CCR5* genotype improves the assessment of AIDS risk in HIV-1-infected individuals**

**Table of Contents**

| ***Section*** | ***Title*** | ***Page*** |
| --- | --- | --- |
| 1 | Classification tree analysis | 2 |
| 2 | Supplementary Figure Legends | 5 |
|  | Figure S1. Classification trees and their clinical application in the WHMC HIV^+^ cohort.  Figure S2. Replication of results of CART analysis in the seroprevalent component of the WHMC HIV^+^ cohort. | 5  7 |
| 3 | References | 8 |
|  |  |  |
|  |  |  |
|  |  |  |

**Section 1. Classification and regression tree (CART) analysis**

Classification trees are commonly used as a method of deductive reasoning for the purposes of data mining and extracting relationships among the predictor variables [1,2,3,4,5]. When the outcome (or the target) variable is categorical in nature, classification trees are used. We thus used a classification tree to predict the AIDS status of a subject. The software program, after pruning through a set of potential candidate trees, chose the tree best fitting the data which is shown in Figure 2A (main text) and Figure S1A. The tree was based on a series of binary diagnostic decisions that best described the cohort data. In the building of the tree, we used following parameters:

| Maximum tree depth | 10 splits |
| --- | --- |
| Minimum node size to split | 10 |
| Method for validating and pruning | 10-fold cross -validation |
| Pruning control times standard error from the mean | 0.5 |
| Variables forced to be included in intial splitting | None |
| Misclassification weight | Equal |
| Imputation of missing data | Yes |
| Variable weights | Equal |

The full tree contained 83 nodes (74 terminal nodes or ‘leaves’) while the final pruned tree contained only 9 nodes (with 5 leaves). This tree was initially generated only from the European- and Hispanic-American component in the WHMC cohort (Figure S1A, N = 690) and was then applied to all seroconverters in this cohort (Figure 2A). Even though the tree was generated only on the basis of a differential risk of developing AIDS, to further assess the robustness of the tree generated, we investigated whether the time to AIDS (1987 criteria) at each nodal split was different (Figure 2C). The results of these analyses revealed that in the seroconverting component of the cohort, each nodal split generated two groups that were statistically significantly different in terms of both the risk of developing AIDS and the rate of progression to AIDS. For generation of the classification trees we used the DTREG (Brentwood, TN) software.

We generated KM plots for the time to AIDS in the subjects belonging to each of the terminal nodes in the final tree. The logrank test (χ^2^ = 60.35, p = 2.4x10^-12^) indicated that the tree generated groups that were significantly different statistically in terms of the rate of progression to AIDS. We also determined the probability of developing AIDS in subjects belonging to each of the terminal nodes. For this we again made use of the concept of prognostic likelihood ratios. Using the overall probability of developing AIDS in the seroconverting cohort (that is without considering any of the three prognostic predictors: viral load, CD4 cell count and GRG status) as a measure of the pre-test probability of AIDS, we estimated the post-test probability of AIDS for subjects belonging to the terminal nodes of the final classification tree. The reason for using likelihood ratios in this setting is to provide a clinical measure in settings where the pre-test probabilities can differ.

To assess the importance of the GRGs in the classification tree, we generated a sub-tree by artificially removing the GRGs as shown and described in Figure 2G-I. This sub-tree had four terminal nodes – i) baseline CD4 < 453 cells/mm^3^; ii) baseline CD4 ≥ 453 cells/mm^3^ and HIV RNA < 17,500 copies/ml; iii) baseline CD4 ≥ 453 cells/mm^3^and HIV RNA 17,500 – <55,500 copies/ml; and iv) baseline CD4 ≥ 453 cells/mm^3^ and HIV RNA ≥ 55,500 copies/ml. We analyzed this sub-tree in the same manner as described above and the results are shown in Figure 2G-I.

**Section 2. Supplementary Figure Legends**

**Figure S1. Classification trees and their clinical application in the HIV^+^ WHMC cohort.** (**A**) A binary tree output for risk of development of AIDS based on baseline CD4 T cell counts (CD4), steady state viral load (VL) and GRG status in the European Americans and Hispanic Americans in the WHMC HIV^+^ cohort. This output was generated using the DTREG program and based on GRG status and the indicated cut-offs for CD4 and VL, the tree identified five exclusive groups designated as A to E. The tree shows that the proximal split was based on the CD4 cell count, and the computer algorithm generated the cut-off point of 453 cells/mm^3^. The next split in the classification tree was based on a viral load of 17,500 copies/ml. The third split was based on GRG status, and then followed by another split at a viral load of 55,500 copies/ml. The misclassification of not having the most likely outcome associated with a terminal node (e.g. AIDS or no AIDS) is indicated. For example, in the proximal-most division, the most likely outcome was AIDS, and this was not achieved in 40.18% of subjects who had a CD4 of <453 cells/mm^3^. The five groups generated are color-coded. (**B** and **C**) Association of the five risk groups generated by this CART strategy with the risk and rate of development of AIDS. For example, Group A in panel A refers to subjects with a CD4 count of less than 453 cells/mm^3^ and the group color-coded as black (Group E) indicates subjects who have a CD4 of ≥453 cells/mm^3^, a low GRG status and a viral load of ≥ 55,500 copies/ml. In the analyses shown, the reference category is group B, which are subjects that have a baseline CD4 of ≥453 cells/mm^3^ and a viral load of <17,500 copies/ml. The risk of development of AIDS was modeled using logistic regression and rate of progression was by Cox proportional hazard models. OR, odds ratio; CI, confidence interval; RH, relative hazard. (**D**) Risk of developing AIDS and (**E**) rate of progression to AIDS in the European and Hispanic American HIV^+^ subjects classified on the basis of the tree shown in panel A but without inclusion of GRG status. The reference category has the same CD4 and viral load characteristics as those in the analyses shown in panels B and C, but does not include the GRGs

**Figure S2. Replication of results of CART analysis in the seroprevalent component of the WHMC HIV^+^ cohort.** (**A**) Prognostic risk groups (Gp) generated by the classification and regression tree (CART) approach in the seroconverting component of the cohort are designated as A to E. VL, viral load. (**B**) KM curves for rates of progression to AIDS, (**C**) relative hazards (RH) of disease progression and (**D**) pre- and post-test probability of development of AIDS in seroprevalent subjects with the indicated CART-generated risk groups. The RH, 95% CI and *P* for rate of progression to AIDS for the CART-generated risk groups A to E are shown in panel C, and the reference group for these analyses (RH = 1) are those with a CD4^+^ cell count greater than 453 cells/mm^3^ and a viral load cut-off of less than 17,500 copies/ml (Group B in panel A). In panel D, “overall” refers to the pre-test probability (Prob) of developing AIDS in the seroconverting adults, and ΔP is the change from this pre-test probability in the CART-generated risk groups. The findings shown in panels A to D are derived from the seroprevalent portion of the cohort and they mirror those found in the seroconverting component of the cohort shown in Figure 2. In panel A, M and H refer to moderate and high GRG, respectively. The color codes in panels B and D correspond to the different groups show in panel A, with the open box referring to the overall study group.

**Section 3. References**

1. Langdorf MI, Rudkin SE, Dellota K, Fox JC, Munden S (2002) Decision rule and utility of routine urine toxicology screening of trauma patients. Eur J Emerg Med 9: 115-121.

2. Lemon SC, Roy J, Clark MA, Friedmann PD, Rakowski W (2003) Classification and regression tree analysis in public health: methodological review and comparison with logistic regression. Ann Behav Med 26: 172-181.

3. Li L, Huang J, Sun S, Shen J, Unverzagt FW, et al. (2004) Selecting pre-screening items for early intervention trials of dementia--a case study. Stat Med 23: 271-283.

4. Province MA, Shannon WD, Rao DC (2001) Classification methods for confronting heterogeneity. Adv Genet 42: 273-286.

5. Vlahou A, Laronga C, Wilson L, Gregory B, Fournier K, et al. (2003) A novel approach toward development of a rapid blood test for breast cancer. Clin Breast Cancer 4: 203-209.

6. Dolan MJ, Kulkarni H, Camargo JF, He W, Smith A, et al. (2007) CCL3L1 and CCR5 influence cell-mediated immunity and affect HIV-AIDS pathogenesis via viral entry-independent mechanisms. Nat Immunol 8: 1324-1336.
