# Supplementary figures and images for: CCL3L1-CCR5 Genotype Improves the Assessment of AIDS Risk in HIV-1-Infected Individuals
Source: PLoS One. 2008 Sep 8;3(9):e3165. doi: 10.1371/journal.pone.0003165 (PMC2522281; doi:10.1371/journal.pone.0003165)

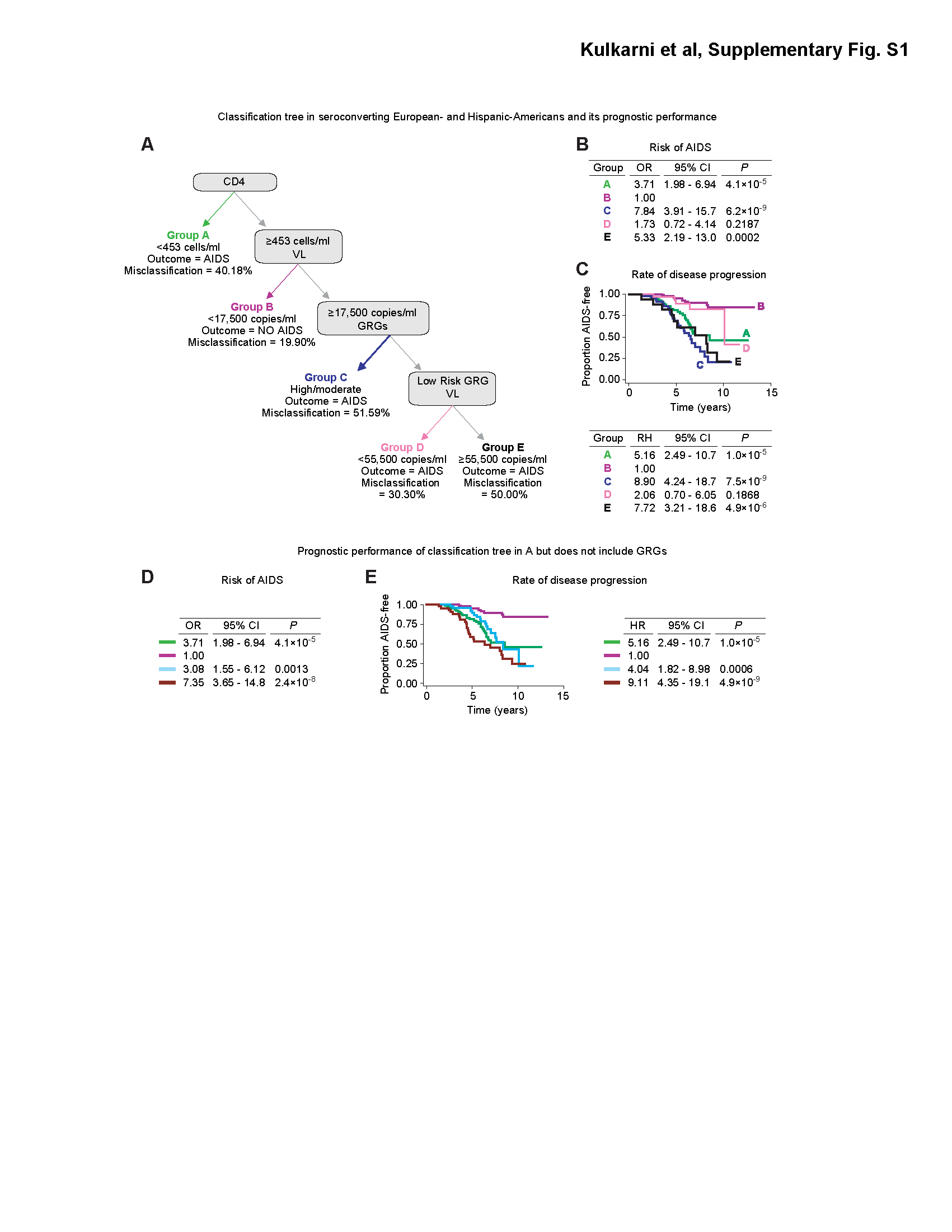

Supplement: Figure S1 — Classification trees and their clinical application in the HIV+ WHMC cohort. (0.35 MB TIF) [file pone.0003165.s002.tif]

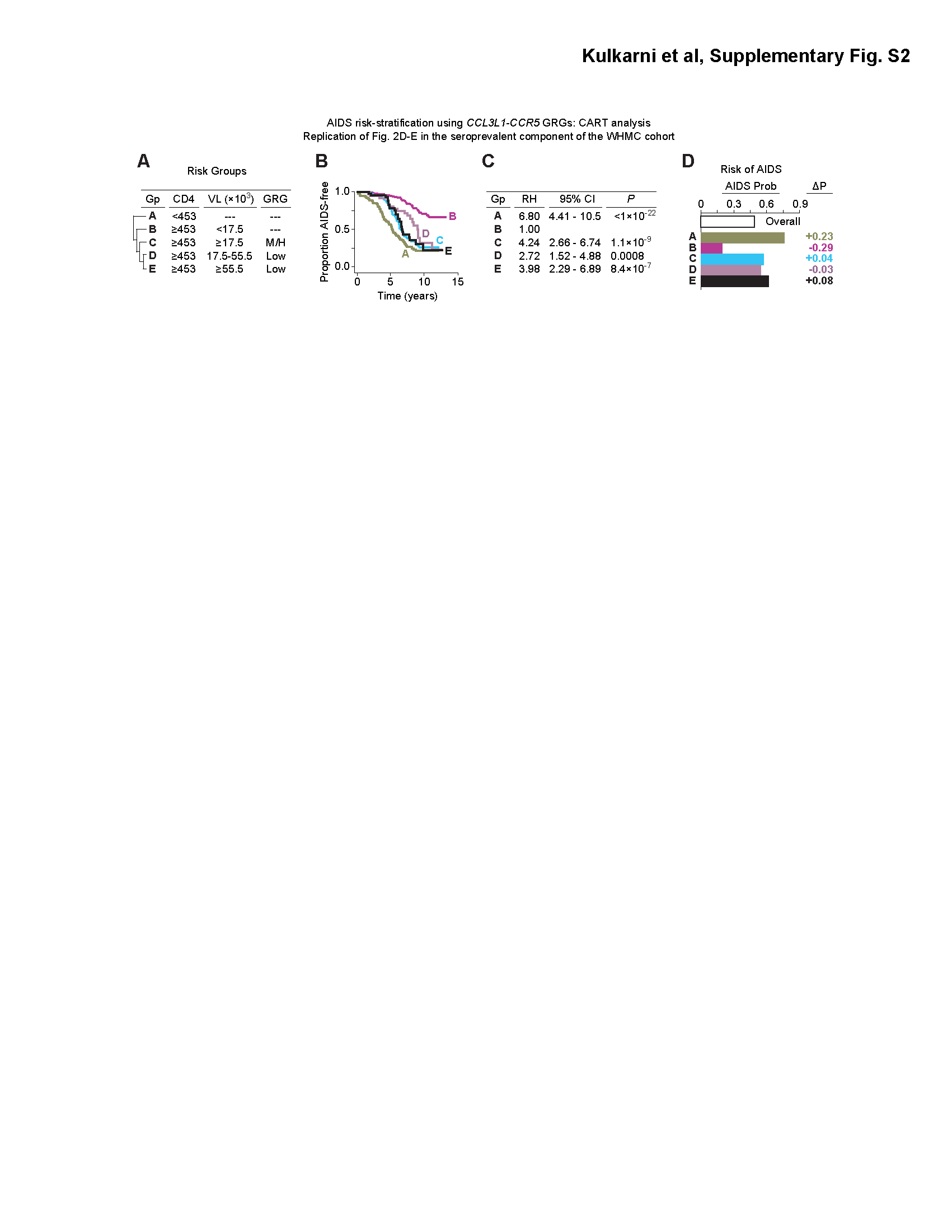

Supplement: Figure S2 — Replication of results of CART analysis in the seroprevalent component of the WHMC HIV+ cohort. (0.31 MB TIF) [file pone.0003165.s003.tif]
