# Supplementary material for: CCL3L1-CCR5 Genotype Improves the Assessment of AIDS Risk in HIV-1-Infected Individuals
Source: PLoS One. 2008 Sep 8;3(9):e3165. doi: 10.1371/journal.pone.0003165 (PMC2522281; doi:10.1371/journal.pone.0003165)
Supplement: Table S1 — (0.04 MB DOC) [file pone.0003165.s004.doc]

**Table S1. Characteristics of the study subjects in the HIV-positive WHMC cohort.**

| Characteristic | Entire cohort | Seroconverters | Seroprevalent* |
| --- | --- | --- | --- |
| N | 1,132 | 515 | 617 |
| Ethnicity (n, %)  European Americans  African Americans  Hispanic Americans  Others ( | 624 (54.9%)  410 (36.3%)  69 (6.1%)  29 (2.7%) | 301 (58.4%)  172 (33.4%)  28 (5.4%)  14 (2.8%) | 323 (52.4%)  238 (38.6%)  41 (6.6%)  15 (2.4%) |
| Maximum length of follow-up (yrs) | 15.47 | 12.83 | 15.47 |
| Total follow-up (person years) | 7123.5 | 2509.9 | 4613.6 |
| Age at cohort entry (mean ± SD yrs) | 29.9 ± 7.2 | 29.3 ± 6.6 | 30.4 ± 7.6 |
| Males (n, %) | 1060 (93.8%) | 489 (95.0) | 571 (92.5) |
| Baseline CD4 cell count (mean ± SE cells/μl) | 605.2 ± 8.96 | 625.1 ± 13.4 | 590.0 ± 12.0 |
| Steady-state viral load (median copies/ml, IQR) | 24000, 66000 | 18000, 44500 | 36000, 81000 |
| Number of subjects who developed AIDS (%) | 450 (39.8%) | 121 (23.5%) | 329 (53.3%) |
| Number of AIDS-related deaths (%) | 440 (38.9%) | 107 (20.8%) | 333 (54.0%) |
| Subjects receiving HAART (n, %) | 375 (33.1%) | 262 (50.9%) | 113 (18.3%) |
| Distribution of *CCL3L1-CCR5* GRGs (n, %)  Low GRG  Moderate GRG  High GRG | 555 (50.3%)  462 (41.9%)  86 (7.87%) | 274 (54.6%)  194 (38.7%)  34 (6.8%) | 281 (46.8%)  268 (44.2%)  52 (9.0%) |

*Seronverters and seroprevalent refer to two groups of subjects from the WHMC HIV^+^ cohort who were followed from the early stages of their infection, albeit the date of seroconversion could be estimated only in the former group [6].
